# Non-contact assessment of cardiac physiology using FO-MVSS-based ballistocardiography: a promising approach for heart failure evaluation

**DOI:** 10.1038/s41598-024-53464-8

**Published:** 2024-02-08

**Authors:** Jing Zhan, Xiaoyan Wu, Xuelei Fu, Chenze Li, Ke-Qiong Deng, Qin Wei, Chao Zhang, Tao Zhao, Congcong Li, Longting Huang, Kewei Chen, Qiongxin Wang, Zhengying Li, Zhibing Lu

**Affiliations:** 1 Hubei Key Laboratory of Broadband Wireless Communication and Sensor Networks, School of Information Engineering, Wuhan University of Technology, Wuhan, 430070 Hubei China; 2 National Engineering Research Center of Optical Fiber Sensing Technology and Networks, Wuhan University of Technology, Wuhan, 430070 Hubei China; 3 State Key Laboratory of Silicate Materials for Architectures, Wuhan University of Technology, Wuhan, 430070 Hubei China; 4 Department of Cardiology, Zhongnan Hospital of Wuhan University, Wuhan, 430071 Hubei China; 5 Institute of Myocardial Injury and Repair, Wuhan University, Wuhan, 430071 Hubei China; 6 State Key Laboratory of Advanced Technology for Materials Synthesis and Processing, Wuhan University of Technology, Wuhan, 430070 Hubei China

**Keywords:** Biological techniques, Diseases, Engineering, Optics and photonics

## Abstract

Continuous monitoring of cardiac motions has been expected to provide essential cardiac physiology information on cardiovascular functioning. A fiber-optic micro-vibration sensing system (FO-MVSS) makes it promising. This study aimed to explore the correlation between Ballistocardiography (BCG) waveforms, measured using an FO-MVSS, and myocardial valve activity during the systolic and diastolic phases of the cardiac cycle in participants with normal cardiac function and patients with congestive heart failure (CHF). A high-sensitivity FO-MVSS acquired continuous BCG recordings. The simultaneous recordings of BCG and electrocardiogram (ECG) signals were obtained from 101 participants to examine their correlation. BCG, ECG, and intracavitary pressure signals were collected from 6 patients undergoing cardiac catheter intervention to investigate BCG waveforms and cardiac cycle phases. Tissue Doppler imaging (TDI) measured cardiac time intervals in 51 participants correlated with BCG intervals. The BCG recordings were further validated in 61 CHF patients to assess cardiac parameters by BCG. For heart failure evaluation machine learning was used to analyze BCG-derived cardiac parameters. Significant correlations were observed between cardiac physiology parameters and BCG's parameters. Furthermore, a linear relationship was found betwen IJ amplitude and cardiac output (r = 0.923, R^2^ = 0.926, *p* < 0.001). Machine learning techniques, including K-Nearest Neighbors (KNN), Decision Tree Classifier (DTC), Support Vector Machine (SVM), Logistic Regression (LR), Random Forest (RF), and XGBoost, respectively, demonstrated remarkable performance. They all achieved average accuracy and AUC values exceeding 95% in a five-fold cross-validation approach. We establish an electromagnetic-interference-free and non-contact method for continuous monitoring of the cardiac cycle and myocardial contractility and measure the different phases of the cardiac cycle. It presents a sensitive method for evaluating changes in both cardiac contraction and relaxation in the context of heart failure assessment.

## Introduction

Cardiovascular diseases are the leading cause of global mortality, and require the continuous monitoring of the cardiac cycle, myocardial motions, and hemodynamic parameters^1^. Accurate assessment of these parameters can provide early detection, evaluation of disease severity, and prognosis assessment of cardiovascular diseases. Among these parameters, cardiac time intervals and cardiac output can accurately reflect the mechanical activity of the heart^2,3^.

Ballistocardiography (BCG) is a non-invasive technique that captures the micro-vibrations induced by heartbeats^4^. BCG signals exhibit synchronized changes with the cardiac cycle and can be obtained without attaching sensors to the skin^5^. However, the interpretation of the BCG recordings and the recognition of their physiological significance have been hindered by the various degrees of distortion and discrepant waveform morphologies, which are observed from the device’s characteristic displays^6–8^. These limitations have hampered progress in BCG research since the 1980s^9^.

Recent technological advancements in biomedical and electrical engineering have brought new possibilities concerning BCG monitoring. Bulky sensing devices that cover the entire body have been replaced by compact sensors embedded in mattresses^10,11^, electronic scales^12,13^, and seats^14^, to enable accurate heart rate monitoring. These compact sensors offer the advantage of convenience and ease of use, potentially reducing the distortion in BCG recordings and revealing more useful information. For instance, Kim et al.^5^ analysis of aortic pressure developed a mathematical model of the BCG signal, which demonstrated a strong association between the genesis of the BCG signal and blood pressure gradients in the ascending and descending aorta. It demonstrated a close correlation between the waveform of BCG and specific events in the cardiac cycle.

In contrast to other non-invasive methods for monitoring cardiac vibrations, such as seismocardiography (SCG)^15^ and gyrocardiography (GCG)^16^, which rely on accelerometers and gyroscope sensors, respectively, the correlation between these signal waveforms and cardiac events has been studied and applied in cardiac function monitoring^17^. However, it is noteworthy that these conventional approaches necessitate attachment to the chest surface. With the advancement of sensors and technology, the concept of non-contact monitoring of cardiac activity has garnered increasing attention due to its numerous advantages over traditional techniques^18^. Non-contact methods encompass the use of radar^19^ smartphones and laptops^20^. Nonetheless, these methods are primarily geared towards monitoring heart rate and may not capture the subtle valve movements within each cardiac cycle.

Therefore, it is crucial to investigate the correlation between the BCG signal and the heart’s actual mechanical motion. Determining this correlation will enhance our understanding of the physiological significance of each sub-wave in the BCG signal, providing a basis for more accurate and convenient cardiac function assessment. Moreover, it lays the foundation for BCG-based non-contact monitoring of cardiac function.

In this study, we aim to investigate the relationship between changes in BCG waveforms, as detected by fiber-optic micro-vibration sensing system (FO-MVSS), and the various phases of the cardiac cycle. By accomplishing this, we intend to advance our understanding of the physiological importance of each BCG sub-wave and establish a foundation for convenient assessment of cardiac function. This research holds promising potential in evaluating heart failure, offering a valuable approach to early detection and intervention.

## Results

### Performance of the FO-MVSS

**Figure 1:**
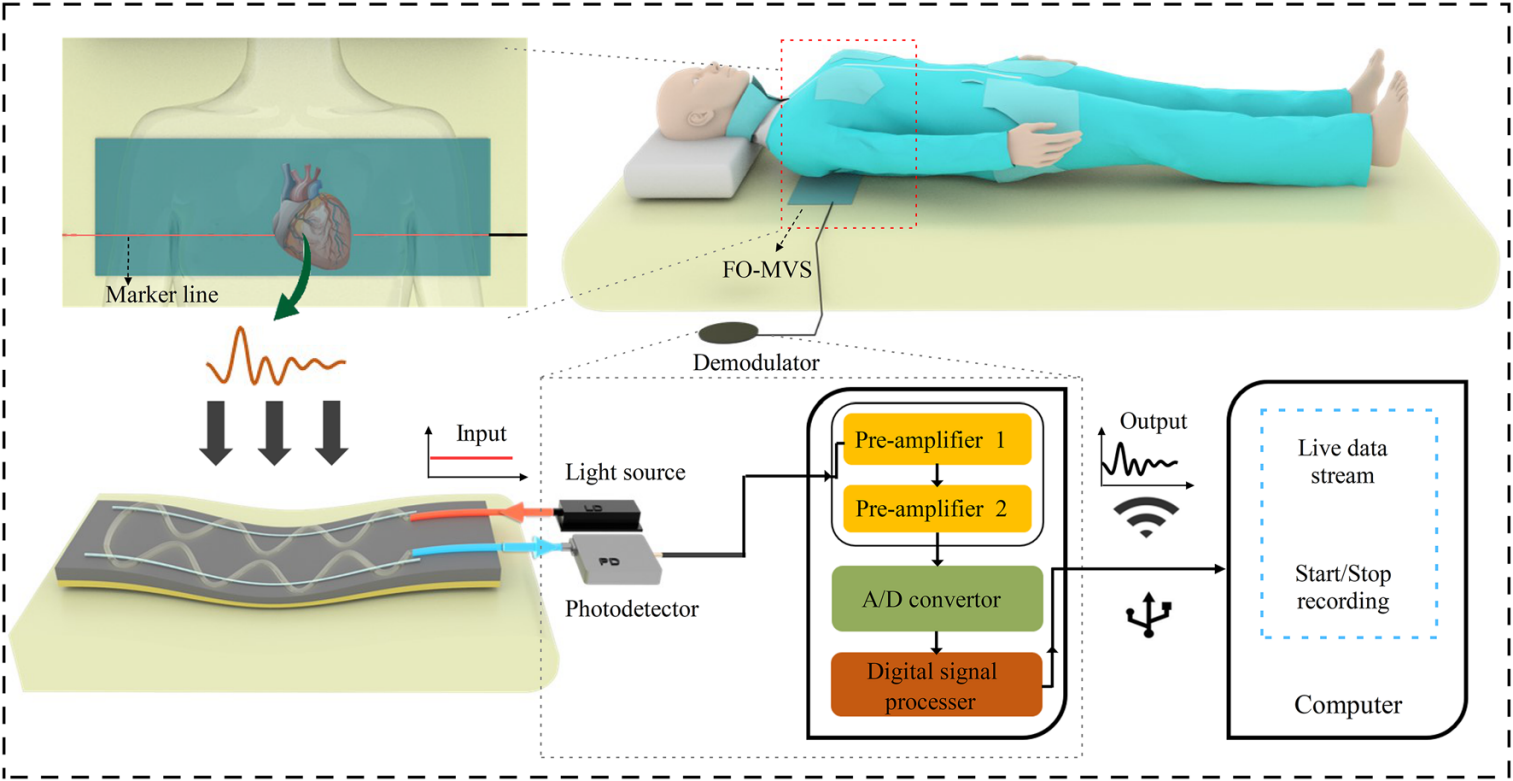
FO-MVSS used to acquire heartbeat-induced vibration signals. The sensor of the FO-MVSS is placed directly below the heart, and the marker line of the FO-MVS is placed along the nipple line or on the fifth intercostal space with the subject in a dorsal decubitus position. The heartbeat-induced vibration signal is detected with FO-MVS, before undergoing optoelectronic conversion by the photodetector, two stages of pre-amplification, A/D conversion, and digital signal processing. A/D, analog-to-digital; FO-MVSS, fiber-optic micro-vibration-sensing system; FO-MVS, fiber-optic micro-vibration sensor.

**Figure 2:**
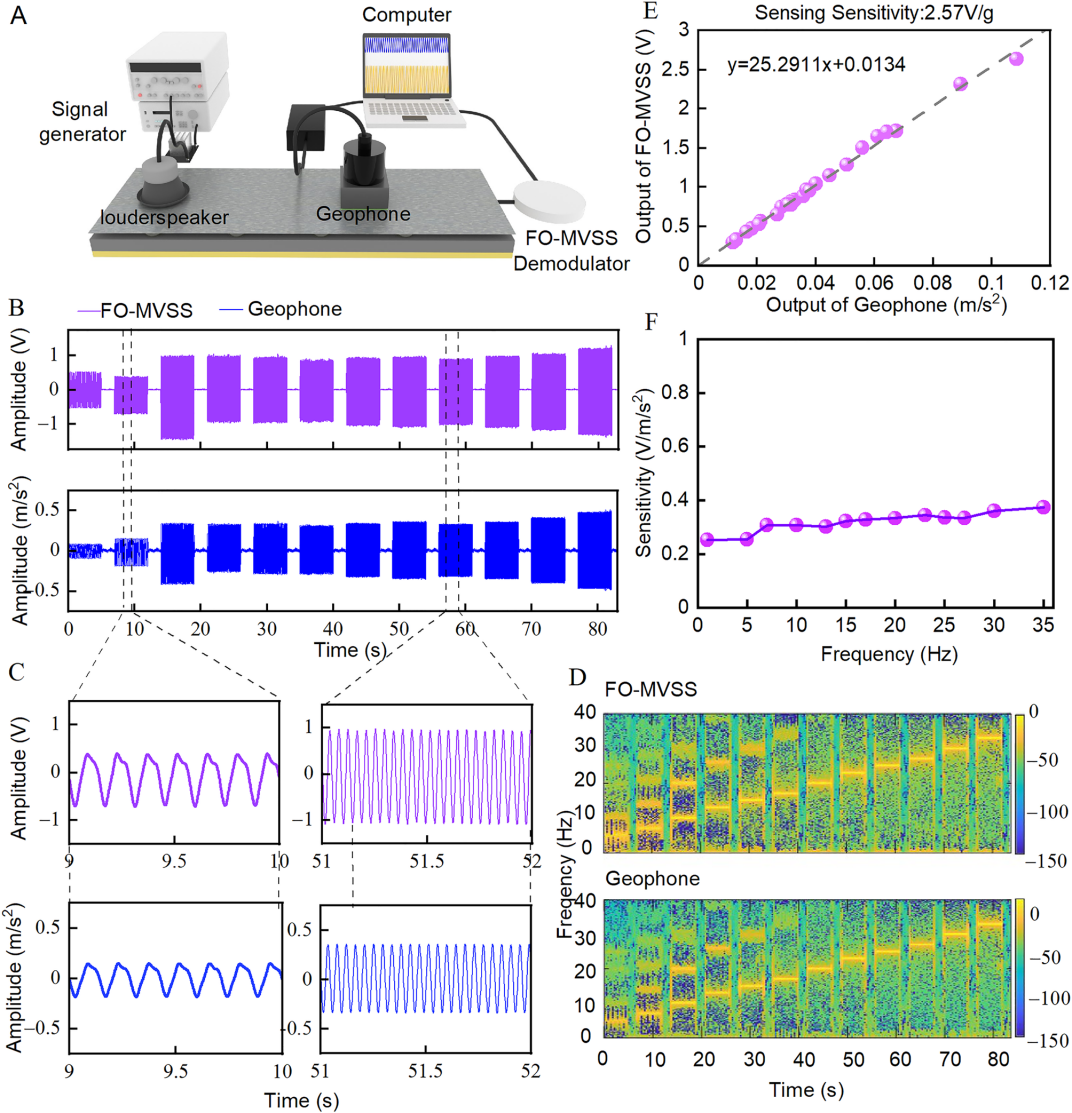
Calibration of the FO-MVSS. (**A**), Schematic illustration of the calibration setup. (**B**), Vibration signals from different frequencies and amplitudes are detected simultaneously by the fiber-optic micro-vibration sensing system (FO-MVSS) and the geophone. C, Zoom-in view of four signal sections in (**B**,**D**), Spectrograms of the signals in B. E, Sensitivity calibration of the FO-MVSS by the geophone, performed at 10 Hz. (**F**), FO MVSS frequency response.

The FO-MVSS (Fig. 1) exhibited a sensitivity of 2.57 V/g at 10 Hz and response variation within 0.51 dB from 0.5 to 35 Hz (Fig. 2). The flat response and relatively stable ground delay in the BCG frequency band effectively reduced the distortion of the recorded signals (Fig. 2). The noise floor was estimated to be 30.1 ± 2.5 mV. According to the FO-MVSS sensitivity ranging from 0.5 to 35 Hz, the vibration resolution of MV-OFSS was 0.115 m/s^2^.

### Verification of the stability of the extracted signal

The respiratory component in the vibration signal was removed via a signal processing algorithm, and a periodic waveform in sync with the heartbeat was obtained (Fig. S1B to S1E). Variation in respiratory rates (12–24, 0, > 24, and < 12 times/min) has minimal influence on the composition of the waveform in the extracted vibration signal (Fig. S1). Each waveform of the BCG had 4 peaks and 3 valleys. Sandbags were placed on the chest or abdomen of the participants. The changes affecting the amplitude and morphology of the waveforms after loading 5-kg or 10-kg weights (Fig. S2) were deemed negligible because the variations in amplitude for each BCG sub-wave were less than 0.02 V.

### Correspondence between BCG recordings and electrocardiograms

**Figure 3:**
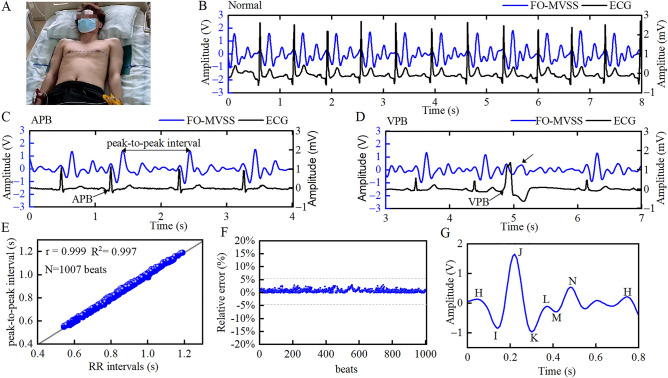
Simultaneously recorded BCG and ECG waveforms. (**A**), A photograph was taken during the experiment. (**B**), BCG, and ECG recordings show a regular heart rate. (**C**), BCG, and ECG recordings showing APBs. (**D**), BCG, and ECG recordings showing VPBs. (**E**), Linear relationship between the RR interval and the peak-to-peak interval. (**F**), The relative error between the heart rate intervals extracted by BCG and the RR intervals measured by ECG. (**G**), A typical BCG waveform. APBs, atrial premature beats; BCG, ballistocardiography; ECG, electrocardiography; FO-MVSS, fiber-optic micro-vibration-sensing system; ICC, intraclass correlation coefficient; VPBs, ventricular premature beats.

When comparing the extracted vibration signal with the synchronously recorded electrocardiogram (ECG), each vibration signal peak appeared consistently with each R peak from the ECG in sinus rhythm (Fig. 3A,B). In the case of an APB observed in the ECG, the corresponding peak in the extracted vibration signal appeared in advance, and was consistent with the atrial premature beat (APB) R peak (Fig. 3C). And in the case of ECG-confirmed ventricular premature beat (VPB), an early and abnormal waveform was observed still in the consistency of time with the VPB R peak (Fig. 3D). This could be linked to the incomplete filling of ventricle failing to produce enough heartbeat vibration. To further verify the time correspondence, a 20-s segment of the extracted vibration signal was randomly sampled from the recording of each subject and compared with the synchronized ECG. The results from 101 participants (Table 1) indicated that the peak-to-peak interval in the extracted signal from the FO-MVSS had a good linear relationship with the RR interval in the ECG, giving a Spearman’s rank correlation coefficient r of 0.999 (*P* < 0.001), and, a coefficient of determination *R*^2^ of 0.997 (Fig. 3E). The relative error in measuring heart intervals using BCG with respect to the measure of RR intervals using ECG was less than 2% (Fig. 3F). These results would suggest that the extracted vibration signal may correspond to the heartbeat and meet the BCG definition. Starr's naming rules^6^ are adopted and the four peaks and three troughs in the waveform are named H, I, J, K, L, M, and N, respectively (Fig. 3G). Similar morphology was observed in the BCG waveforms of participants with normal cardiac function, all composed of peaks and troughs from H to N, irrespective of sex, age, height, and weight. Each R peak time of ECG had a good linear relationship with the H peak time of each BCG, with a Spearman’s rank correlation coefficient r of 0.965 (*P* < 0.001), and, a coefficient of determination *R*^2^ of 0.999.

**Table 1:** The characteristics of 101 participants with normal cardiac function (NCF).

Characteristics	NCF (n = 101)
Age, y	$49.11\pm 29.28$
Male sex, n,	55%, 55
Height, cm	$170.21\pm 8.05$
Weight, cm	$65.68\pm 11.32$
BMI, Kg/m^2^	$22.33\pm 3.07$
Heart rate, beats/min	$73.02\pm 12.19$
LAD, mm	$32.78\pm 2.98$
LVEDD, mm	$47.53\pm 4.64$
EDV, ml	$107.04\pm 27.35$
ESV, ml	$35.39\pm 12.26$
SV, ml	$70.43\pm 20.28$
CO, L/min	$5.12\pm 1.66$
EF, %	$67.39\pm 7.33$

BMI, body mass index; HR, heart rate; LAD, left atrial diameter; LVEDD, left ventricular end of diastole diameter; EF, ejection fraction;

### BCG-based cardiac cycle phases

**Figure 4:**
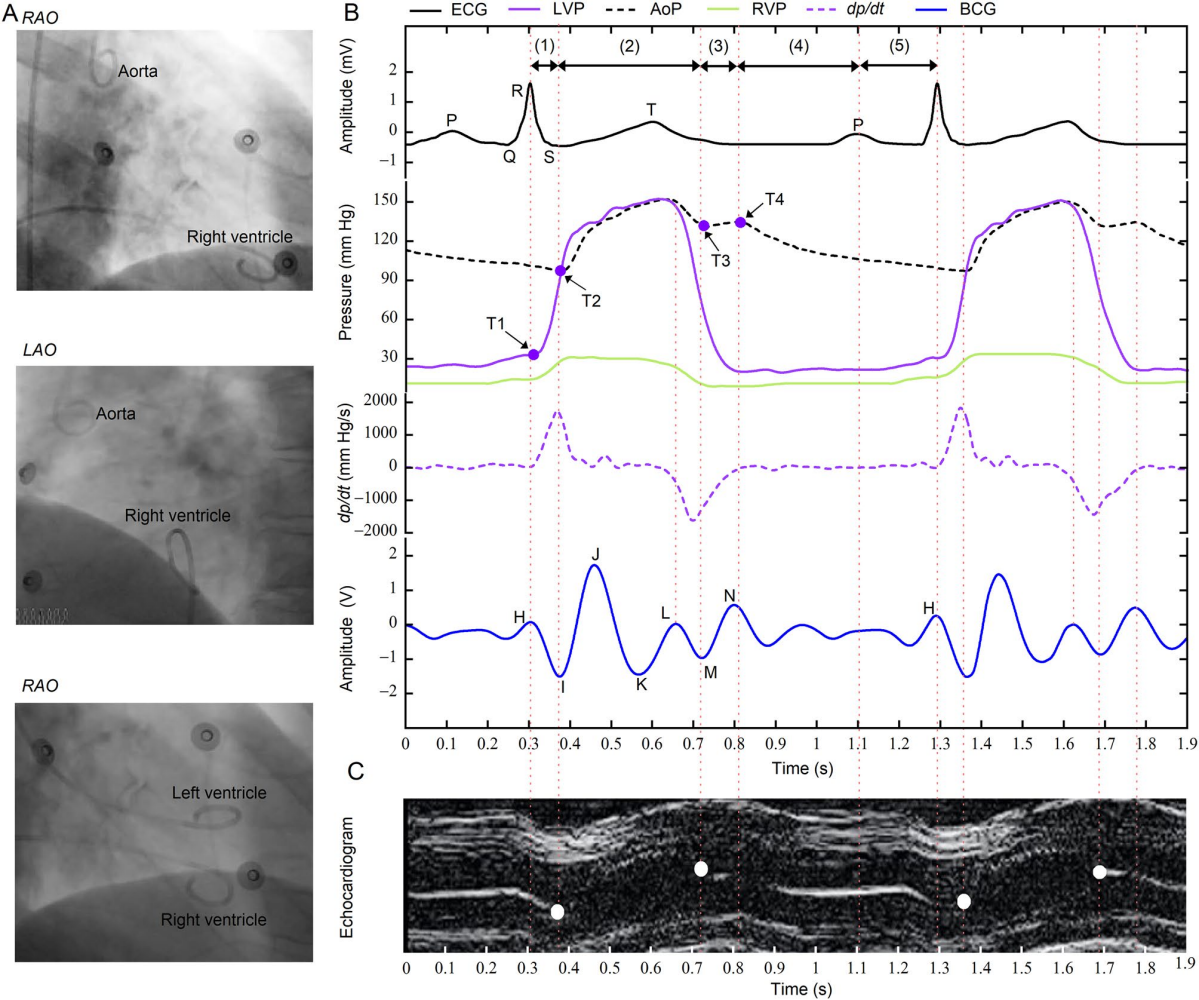
Correspondence between the sub-waves of the BCG waveform and the phases of the cardiac cycle. The ECG recordings, intracardiac pressure curves, and M-mode echocardiograms were synchronously obtained for verification. (**A**), Location of the intracardiac catheters. (**B**–**C**), Synchronized electrocardiogram with LVP, AoP, RVP, and dp/dt (rate of change in LVP) curves; BCG recording; and M-mode echocardiogram of the aortic valve of one subject.AoP, aortic pressure; BCG, ballistocardiography; LAO, left anterior oblique; LVP, left ventricular pressure; RAO, right anterior oblique; RVP, right ventricular pressure.

The BCG and ECG recordings, intracardiac pressure curves, and M-mode echocardiograms were simultaneously obtained from 6 patients. For proper illustration, the synchronized results from one subject are shown in Fig. 4. Each sub-wave of the BCG waveform corresponded to a certain phase of the cardiac cycle (Fig. 4B,C). The peak of the H wave (the H point) was aligned with the T1 point (start of the isovolumetric contraction phase) in the LVP curve, which also represented the moment of mitral valve closing. The trough or the I wave (the I point) corresponded to the T2 point (start of the left ventricular ejection phase), the maximum of *dp*/*dt* (change rate of the left ventricular pressure), and the moment of aortic valve opening. The peak of the J wave (the J point) corresponded to the notch in the ascending segment of the left ventricular pressure curve, and *dp*/*dt* drops to around 0. The peak of the L wave (the L point) matched the turning point when the ventricular pressure starts to decline from the highest point. The trough of the M wave (the M point) corresponded to the T3 point (beginning of the isovolumetric relaxation phase) and the timing of the aortic valve closure. The peak of the N wave (the N point) corresponded to the T4 point (beginning of the filling phase) and the timing of the mitral valve opening. The sub-wave from the N point to the next H point corresponded to the phases from the ventricular filling to the end of atrial contraction. The BCG recordings from the other 5 participants presented the same similitudes with the events in a cardiac cycle.

Based on the experimental findings, we proposed a method for the categorization of BCG based on the phases of the cardiac cycle (Table 2). The H point could correspond to the timing of the mitral valve closure, while the interval from H to I could represent the isovolumetric contraction phase. At the I point, the aortic valve opening could mark the transition to the left ventricular ejection phase, which may extend from I to M. The M point could refer to the closure of the aortic valve, and the interval from M to N may indicate the isovolumetric relaxation phase. From the N point until the subsequent H point, the cardiac cycle enters diastole, when synchronized with the ECG, the cardiac diastole could be further subdivided into two stages: the ventricular filling phase and the atrial contraction phase.

**Table 2:** Relationship between the sub-waves of the BCG waveform and phases of the cardiac cycle.

Cardiac cycle	Critical point	Physiological event	Physiological significance
Systole	H	Mitral valve closing	HI interval: IVCT
	I	Aortic valve opening	IJ interval: rapid ejection period
	J	End of fast ejection	JM interval: slow ejection period
	K	─	IM interval: LVET
	L	LVP starts to drop	I point: the moment of *dp*/*dt*_max_
	M	Aortic valve closing	
Diastole	N	Mitral valve opening	MN interval: IVRT NH interval: ventricular filling and atrial contraction
	H	Mitral valve closing	

IVCT, isovolumetric contraction time; LVET, left ventricular ejection time; IVRT, isovolumetric relaxation time.

### Cardiac time intervals measured by the BCG-based cardiac cycle phases categorization and tissue Doppler imaging

**Figure 5:**
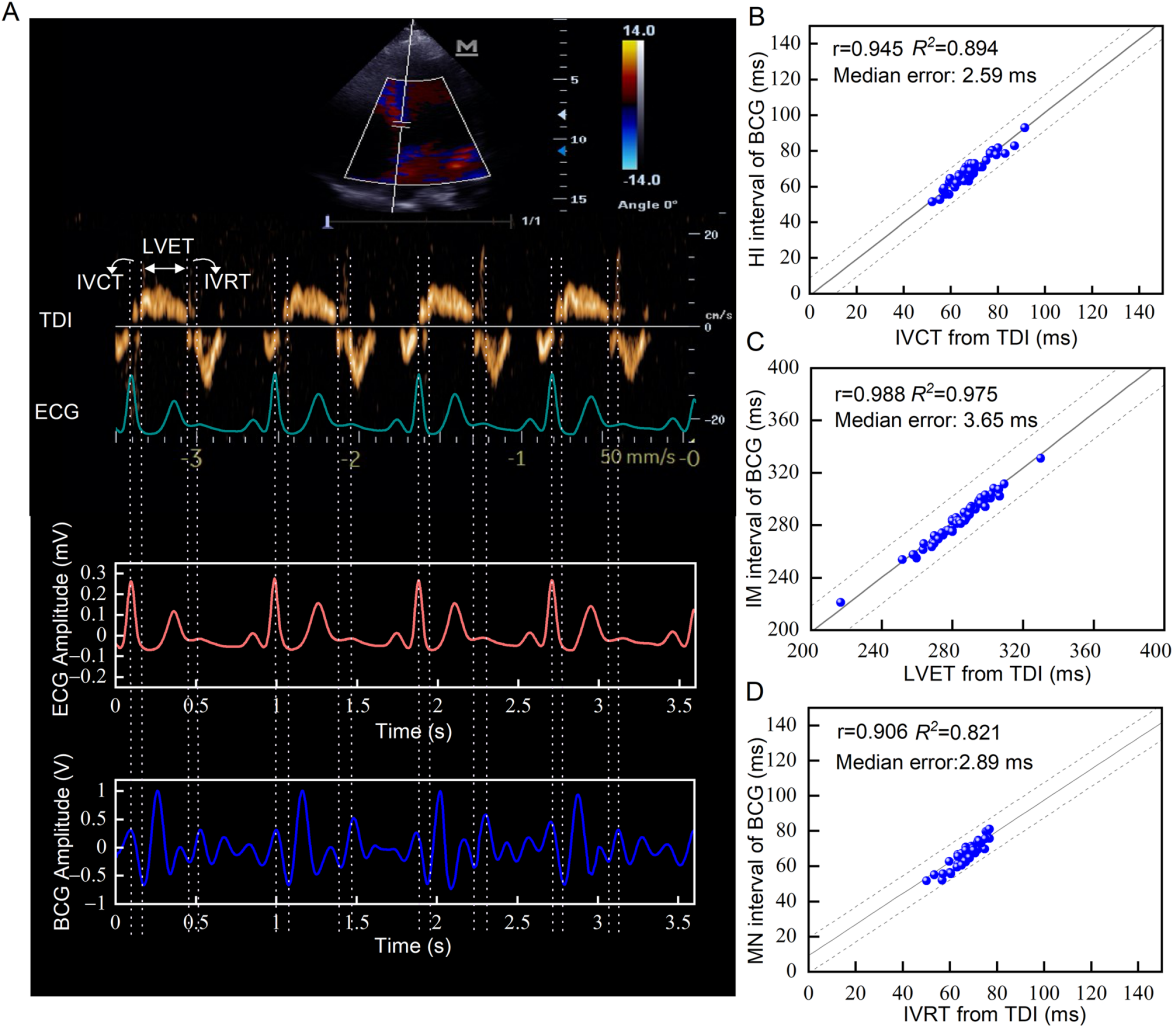
Time parameters measured by BCG and TDI. (**A**), The IVCT, LVET, and IVRT measured by TDI and correspondingly obtained from the BCG recordings. (**B**), Correlation between the HI interval and the IVCT. **C,** Correlation between the IM interval and the LVET. (**D**), Correlation between the MN interval and the IVRT. BCG, ballistocardiography; IVCT, isovolumetric contraction time; IVRT, isovolumetric relaxation time; LVET, left ventricular ejection time; TDI, tissue Doppler imaging.

According to the similitudes observed between the BCG waveform and the cardiac cycle, it would be reasonable to use the HI, IM, and MN intervals in the BCG waveform, respectively to estimate the isovolumetric contraction time (IVCT), left ventricular ejection time (LVET), and isovolumetric relaxation time (IVRT). These hemodynamic parameters measured by BCG were compared with those measured by tissue Doppler imaging (TDI)^21^ in echocardiography for 51 participants with normal cardiac function (Table S1). Compared with the IVCT measured by TDI, the HI interval measured by BCG yielded a Spearman’s rank correlation coefficient r of 0.945 (*P* < 0.001), and a coefficient of determination *R*^2^ of 0.894, with a median absolute error as low as 2.59 ms (Fig. 5B). The results from the IM and MN interval measurements also highly correlated with the LVET and IVRT respectively as measured by TDI (Fig. 5C,D). The IM interval measured with BCG gave a Spearman’s rank correlation coefficient r of 0.988 (*P* < 0.001), and a coefficient of determination *R*^2^ of 0.975, with a median absolute error as low as 3.65 ms. And the MN interval measured by the BCG approach had a Spearman’s rank correlation coefficient r of 0.906 (*P* < 0.001), and a coefficient of determination *R*^2^ of 0.821, with a median absolute error as low as 2.89 ms.

### Relationship between IJ amplitude and myocardial contractility

Considering the correspondence between the IJ interval and the ventricular ejection phase, we further investigated the relationship between the IJ amplitude and the myocardial contractility^21^. The IJ amplitudes of 11 participants with normal cardiac function (Supplementary Table S2) at resting state and after exercise were obtained, and compared to the CO measured by echocardiography. The IJ amplitude was found to increase after exercise in each of the 11 cases (Fig. S3A to S3C). The increase in the IJ amplitude (ΔIJ) and the increase in the cardiac output (ΔCO) (CO) presented a linear positive relationship, with a Spearman’s rank correlation coefficient r of 0.9227 (*P* < 0.001)*,* and*, a* coefficient of determination* R*^*2*^ of 0.8721 (Fig. S3D).

### The usefulness of cardiac time intervals and amplitude parameters by BCG-based cardiac cycle phases categorization

**Figure 6:**
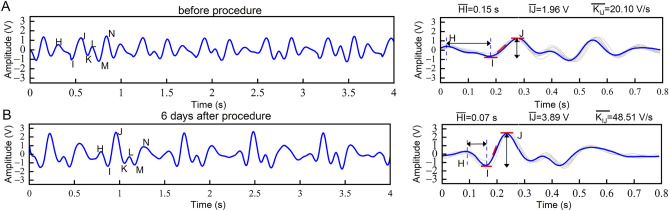
Comparison of the BCG waveforms in patients with CHF before and after treatments. (**A**-**B**), The BCG waveforms before the procedure and 6 days after implantation of CCM in a patient with CHF, the HI intervals decreased from an average of 0.15 s before the procedure to 0.07 s on the 6^th^ day after the procedure. The IJ amplitude increased from 1.96 V to 3.89 V and the waveform morphology progressively restored to normal after the procedure. The average IJ slope increased notably from an average of 20.10 V/s before the procedure to 48.51 V/s on the 6th day. $\overline{{{\text{IJ}}}}$: average IJ amplitude; $\overline{{{\text{HI}}}}$: average HI interval; $\overline{{{\text{K}}_{{{\text{IJ}}}} }}$: average IJ slope.

The usefulness of cardiac time intervals and amplitude parameters by BCG-based cardiac cycle phases categorization. We analyzed the distribution ranges of BCG-derived feature parameters from a cohort of 101 participants with normal cardiac function or control and 61 patients with heart failure (Table 3). In the heart failure group, we observed significant changes in the cardiac time intervals and amplitude parameters derived from BCG signals. More precisely, the HI interval (corresponding to IVCT) showed a significant increase in duration and increased from 65 ± 12 ms to 102 ± 32 ms (*p* < 0.001) as compared to the one from the healthy cohort. The IM interval (corresponding to IVET) was decreased among the heart failure group, with values changing from 310 ± 33 ms to 260 ± 37 ms (*p* < 0.001) compared to the group with normal cardiac function. While the MN interval (corresponding to IVRT) was increased in duration, from 69 ± 12 ms to 93 ± 25 ms (*p* < 0.001) when compared to the control group. Regarding the parameters’ amplitude, notable variations were observed (Table 4). The IJ amplitude (2.715 ± 0.716 mV to 2.163 ± 0.803 mV *p* < 0.001), which has previously been correlated with cardiac output, showed a significant decrease in the heart failure group. In contrast, both the HI amplitudes (0.900 ± 0.219 mV to 1.286 ± 0.497 mV *p* < 0.001) and MN amplitudes (1.160 ± 0.480 mV to 1.405 ± 0.695 mV *p* < 0.001) were significantly larger in the heart failure group compared to the normal cardiac function group (control group). As for the slope parameters, the IJ slope (35.707 ± 9.026 to 27.916 ± 11.586 *p* < 0.001) demonstrated an evident decrease in magnitude with the onset of heart failure compared to the control group. By continuously acquiring the BCG recording before and after the CCM implantation, the changes in the BCG recordings were consistent with the symptom relief, the reduction of the left ventricular end-diastolic diameter, and an increase in EF (Fig. 6A,B).

**Table 3:** The characteristics of patients with heart failure.

Characteristics	CHF (n = 61)
Age, y	$62.13\pm 14.27$
Male sex, n,	44%, 27
Height, cm	$162.74\pm 6.34$
Weight, cm	$63.16\pm 11.86$
BMI, Kg/m^2^	$23.78\pm 3.88$
Heart rate, beats/min	$79.52\pm 16.61$
LAD, mm	$42.82\pm 7.88$
LVEDD, mm	$55.74\pm 9.47$
EDV, ml	$190.14\pm 79.81$
ESV, ml	$110.46\pm 59.84$
SV, ml	$76.96\pm 30.15$
CO, L/min	$6.15\pm 2.95$
EF, %	$40.26\pm 6.59$

BMI, body mass index; HR, heart rate; LAD, left atrial diameter; LVEDD, left ventricular end of diastole diameter; EF, ejection fraction;

**Table 4:** The distribution of cardiac time intervals and amplitudes parameters measured by BCG in the control group and CHF patients.

Characteristics	Control group (n = 101)	CHF (n = 61)	P Value
HI interval (ms)	$65\pm 12$	$102\pm 32$	*P* < 0.001
IM interval (ms)	$310\pm 33$	$260\pm 37$	*P* < 0.001
MN interval (ms)	$69\pm 12$	$93\pm 25$	*P* < 0.001
HI amplitude (mV)	$0.900\pm 0.219$	$1.286\pm 0.497$	*P* < 0.001
IJ amplitude (mV)	$2.715\pm 0.716$	$2.163\pm 0.803$	*P* < 0.001
MN amplitude (mV)	$1.160\pm 0.480$	$1.405\pm 0.695$	*P* < 0.001
HI slope	$-18.450\pm 6.530$	$-15.018\pm 9.364$	0.013
IJ slope	$35.707\pm 9.026$	$27.916\pm 11.586$	*P* < 0.001
MN slope	$13.161\pm 5.110$	$15.733\pm 6.969$	0.011

HI interval, the time interval from the H point to the I point of BCG, which corresponds to the IVCT; IM interval, the time interval from the I point to the H point of BCG, which corresponds to the LVET; MN interval, the time interval from the M point to the N point of BCG, which corresponds to the IVRT; HI amplitude, the amplitude from the H point to the I point of BCG; IJ amplitude, the amplitude from the I point to the J point of BCG; MN amplitude, the amplitude from the M point to the N point of BCG; HI slope, the slope from the point H to the I point of BCG; IJ slope, the slope from the point I to the point J of BCG; MN slope, the slope from the point M to the point N of BCG.

### Performance evaluation of BCG-derived cardiac time and amplitude parameters for heart failure Classification

The performance evaluation of BCG-derived cardiac time and amplitude parameters for heart failure classification involved training and evaluating six distinct classification models: Support Vector Machine (SVM), k-Nearest Neighbors (KNN), Decision Tree Classifier (DTC), Logistic Regression (LR), Random Forest (RF), and XGBoost. Employing a five-fold cross-validation approach, we assessed the average accuracy, AUC, sensitivity, specificity, and F1 score of each model. The specific results are presented in Table 5, showcasing that all six classifiers achieve accuracy levels surpassing 95%, with logistic regression demonstrating superior overall performance compared to the other models. This outcome highlights the capability of the nine parameters extracted through BCG to precisely capture cardiac pulsations and predict cardiac functionality.

**Table 5:** Comparative analysis of average accuracy, average AUC values, average specificity, average sensitivity, and average F1 score among various classification models in the assessment of heart failure using fivefold cross-validation.

Metrics	LR	SVM	KNN	DTC	RF	XGBoost
Accuracy	0.989 ± 0.01	0.983 ± 0.01	0.974 ± 0.01	0.972 ± 0.02	0.970 ± 0.03	0.976 ± 0.02
AUC value	0.986 ± 0.01	0.982 ± 0.01	0.960 ± 0.02	0.963 ± 0.02	0.964 ± 0.03	0.972 ± 0.02
Specificity	0.993 ± 0.01	0.985 ± 0.03	0.990 ± 0.01	0.982 ± 0.01	0.978 ± 0.02	0.980 ± 0.02
Sensitivity	0.979 ± 0.02	0.979 ± 0.03	0.929 ± 0.03	0.944 ± 0.04	0.950 ± 0.05	0.965 ± 0.02
F1 score	0.979 ± 0.02	0.969 ± 0.02	0.949 ± 0.03	0.947 ± 0.04	0.944 ± 0.05	0.955 ± 0.04

LR, Logistic regression. SVM, support vector machines; KNN, K-nearest neighbor;DTC, Decision tree RF, Radom forest. XGBoost, Extrme gradient boosting. AUC, area under the curve.

## Discussion

In this study, several key innovations in the field of cardiac monitoring are presented. Firstly, BCG recordings were successfully obtained using the high-sensitivity FO-MVSS, enabling non-contact and continuous monitoring of cardiac motion. Secondly, our study demonstrated that BCG based on FO-MVSS may accurately determine the key phases of the cardiac cycle. The duration of isovolumetric contraction, ejection, and isovolumetric relaxation could be assessed. Thirdly, the cardiac time intervals and amplitude parameters extracted from the BCG-based cardiac cycle categorization method demonstrate consistent changes in the clinical presentation of CHF patients, indicating its potential in the monitoring of heart failure progression. These findings could collectively contribute to the advancement of non-contact cardiac monitoring techniques.

### FO-MVSS-based BCG and the cardiac motion

The BCG recordings were obtained using an FO-MVSS with high sensitivity and resolution. The vibration sensing device fiber-optic micro-vibration sensor (FO-MVS), which was recently developed in our center^22^ guaranteed the safety of the user as it did not require direct contact with the human body. The FO-MVSS presented a flat response and relatively stable ground delay in the BCG frequency band (0.5—35 Hz), thus, warranted the accuracy of signal acquisition (Fig. S5). The high resolution ensured the FO-MVSS’s ability to capture vibrations 80 times weaker than the gravitational acceleration. It was verified that the BCG recordings obtained by the FO-MVSS would present excellent stability and consistency in participants with normal cardiac function. By implementing the signal processing algorithms including de-trending and multiple resolution reconstruction via discrete wavelet transform, the vibration signal components resulting from the respiratory motion and muscle fibrillation were removed. The results indicated that the BCG waveforms could exhibit similar morphology at different respiratory rates for the same subject (Fig. S1). Meanwhile, the BCG waveforms changed only slightly after loading 5-kg or 10-kg sandbags on the chest or abdomen of the same subject (Fig. S2). This insensitivity to loadbearing may be attributed to the fact that the BCG recordings originate from the alternating current of the acquired signal, but the added load affected mainly the direct current component. Furthermore, the FO-MVS was placed directly below the heart. Thus, the vibration was closely related to cardiac mechanical activity. As such, the morphology and amplitude of the BCG recordings were not greatly affected by loadbearing (Fig. S2). The BCG recordings were reliable, as demonstrated by the peaks and troughs from H to N being consistently observed in the waveforms of participants with normal cardiac function, regardless of sex, age, height, and weight.

### The BCG-based cardiac cycle phases categorization

The experimental results synchronously obtained by electrocardiography, BCG, intracardiac pressure measurement, and M-mode echocardiography showed that the BCG waveforms presented one-to-one correspondence with the cardiac mechanical activity from sinus rhythm. The relationship between the sub-waves in each BCG waveform and the cardiac pumping activities during the systolic and diastolic phases of a cardiac cycle was established. Compared to BCG waveforms obtained by previous devices (Fig. S6)^6^, the physiological significance of each sub-wave in the BCG waveforms was thoroughly explained (Table 2). The H point marks the beginning of the isovolumetric contraction phase of a cardiac cycle when the LVP starts to rise. The rate of LVP rises to reach the maximum (*dp*/*dt*_max_) at the I point, which represented the end of the isovolumetric contraction phase and the beginning of the ejection phase. The J point corresponds to the moment of *dp*/*dt* decreasing to nearly zero. Hence, the J point may help to accurately distinguish the fast and the slow ejection phases. The M point represents the beginning of the isovolumetric relaxation phase. As the rate of the LVP decreases to 0 at the N point, the isovolumetric relaxation phase ends and the ventricular filling phase begins. The interval between the N point and the next H point (NH interval) could be divided into the ventricular filling phase and the atrial systolic phase using the peak of the P wave from the synchronized ECG. The time parameters (IVCT, LVET, and IVRT) in a cardiac cycle measured by BCG (HI, IM, and MN intervals) were highly consistent with the results measured by TDI in echocardiography. Overall, the BCG recordings can effectively identify every specific event from cardiac contraction to relaxation^23^.

### BCG and myocardial contractility

It was verified that the variation of IJ amplitude (ΔIJ) was positively correlated with ΔCO (Fig. S3). As the CO of the individuals increased notably after exercise, the IJ amplitude increased accordingly. A large IJ slope (K_IJ_) was associated with a faster pump speed, which might imply stronger myocardial contractility^24^. The HI interval measured by BCG was highly consistent with the IVCT measured by classical means such as TDI (Fig. 5). A shorter HI interval indicated that the ventricular pressure reached the maximum rising rate in a shorter time, implying greater myocardial contractility^24^.

To verify the effectiveness of BCG monitoring and determining cardiac function, the BCG waveforms of one patient diagnosed with CHF were continuously monitored before and after CCM implantation. Changes in the BCG waveform morphology and time intervals matched with the common clinical indicators of cardiac function. The amplitude of the BCG waveform was higher after CCM implantation than before CCM implantation, the waveform morphology returned to near-normal. The increased IJ amplitude, the shorter HI interval, as well as the increased IJ slope, were consistent with the relief of clinical symptoms and the improvement in echocardiographic parameters.

### Potential application of BCG-based cardiac cycle phase categorization

A study conducted by Biering-Sørensen^25^ supports the usefulness of cardiac time intervals by TDI in predicting cardiac dysfunction, particularly IVCT and the myocardial performance index (MPI) which was computed with cardiac time intervals as independent predictors of heart failure. The cardiac time intervals obtained by BCG in our studying showed consistent changes in the heart failure group as indicated by ultrasound measurements. It is noteworthy that BCG may enable a more convenient and comfortable long-term monitoring experience, as the sensor does not require any contact with the skin. This makes it suitable for prolonged monitoring in both clinical and home settings. In addition, the substantial differences in BCG waveform amplitudes among the heart failure and the control group indicated that BCG waveforms could reflect cardiac mechanical activity. Changes in waveforms may provide insights into the functional status of the heart.

### Study limitations

One of the limitations of the proposed method relied on the requirement for the subject to remain calm during the monitoring process because of the high sensitivity to vibrations. Therefore, participants who could not cooperate, such as those with Parkinson's syndrome, were excluded from the study. Another limitation was that the BCG recordings were obtained under the combined activities from all the cardiac chambers and large vessels. The influences of the left and the right ventricles, as well as the large vessels, could not be differentiated. However, as verified by the recorded LVP, RVP, and AoP (Fig. 4), the BCG waveforms mainly reflected the mechanical contraction of the left ventricle. Moreover, further research is needed to validate the reliability and effectiveness of these BCG-derived parameters in larger cohorts and diverse populations. Standardization of BCG signal acquisition and analysis protocols would also be beneficial. Nevertheless, this work is still in ongoing progress.

## Conclusion

In this study, we advanced BCG recording and proposed a novel method for cardiac cycle phase categorization. By utilizing BCG, we measured cardiac interval time and correlated amplitude parameters with hemodynamic parameters. This approach could enable real-time monitoring of heart rhythm, cardiac function, and potential prediction of heart failure. Our findings highlighted the clinical value of BCG-based cardiac cycle phase categorization, offering a non-contact and convenient cardiac functional assessment. This method has the potential to revolutionize cardiology by improving patient care through timely detection and treatment implementation. Our research contributes to the understanding and implementation of BCG in cardiac monitoring, paving the way for future investigations and validation studies attached to this area.

## Methods

### Study population and protocol

The data supporting the findings of the research can be obtained upon reasonable request from the corresponding authors. There were 111 participants with normal cardiac function recruited, and 61 patients with CHF were recruited, all participants were in sinus rhythm. All participants gave written informed consent, and the study was performed in accordance with the second Helsinki Declaration and approved by the institutional review board of Zhongnan Hospital of Wuhan University (2,022,075).

### Cardiac signals acquisition and stability analysis

#### Fibre-optic micro-vibration sensor

The fiber-optic micro-vibration sensor (FO-MVS) was a mat embedded with a macro-bending optical fibre sensor, which was proposed for non-contact vital signs monitoring in our previous work^22^. The mat is designed as a sandwich structure for encapsulate the FO-MVS for stable sensing under complicated conditions. The whispering gallery mode in a bent single-mode optical fibre was utilized by the FO-MVS to detect weak vibration signals. High vibration sensitivity was obtained by optimizing the fibre radius (110 υm)^22^.

#### FO-MVSS calibrations

The calibration platform is presented in Fig. 2. The vibration signal (0.5–35 Hz) generated by a loudspeaker was detected simultaneously and respectively by the FO-MVSS (V) and a geophone (Jiangsu China DONGHUA610V, m/s^2^).

#### Sensitivity measurement

The sensitivity of a vibration sensor is defined as the ratio of the output voltage to acceleration (U/a)^26^. It quntifies the sensor's ability to detect and convert vibrations into measurable electrical signals. As illustrated in Fig. 2, a dynamic calibration system was employed to calibrate the vibration sensitivity of the FO-MVSS. In Fig. 2E, at a vibration signal frequency of 10 Hz, the output voltage of the FO-MVSS exhibited a monotonically increases with rising acceleration (obtaining from the geophone) achieved by elevating the intensity of the vibration signal generated by loudspeaker. Subsequently, we calculated the ratio of the output voltage to acceleration, yielding the vibration sensitivity of FO-MVSS.

#### Frequency response measurement

The sensitivity and frequency responses from the FO-MVSS were obtained by calibration with the output of Pre-amplifier 2 and that of the geophone.This calibration process facilitated the assessment of the FO-MVSS’s amplitude and the frequency response characteristics.Simultaneously, the signals acquired by the FO-MVSS and the excitation signals supplied to the loudspeaker were fed into an oscilloscope, this arrangement allowed for the precise measurement of phase variation in the sensing signal when subjected to a range of excitation signal frequencies (0.5-35 Hz).

#### Vibration resolution

Vibration resolution is determined by averaging the noise floors from 7 different demodulation controllers,measured during 1-h runs without load. The vibration resolution is determined by the ratio of the noise floor to the sensitivity of the FO-MVSS.

#### Data transmission

Data could be transmitted to the computer via a virtual serial port or wireless connection.

#### Digital signal processing

The BCG recordings were acquired using discrete wavelet transform. Each 8-s sequence of the original signals was decomposed into 11 sub-components through the Symlet wavelet filter with four vanishing moments (sym4)^27,28^. Subsequently, the BCG waveform was obtained by synthesis of the 5^th^ to 10^th^ subcomponents.

#### Acquisition of the BCG recordings

The subject was requested to lie flat on the FO-MVS^22^, which is a mat placed under the mattress of a bed (“non-contact” means that the sensor does not direct contact with the body), and the marker line of the sensor was placed along the nipple line or on the 5^th^ intercostal line (Fig. 1). During the BCG collection, the subject was to stay relaxed and calm.

#### BCG recording acquisition at different respiratory rates

The BCG recordings were obtained from 10 participants with normal cardiac function. During the collection, the participants were respectively asked to breathe normally, fast, slow, and hold their breath. It was ensured that each group of signals contained all four respiratory states, to observe the influence of the respiratory rate on the BCG waveform morphology.

#### Collection and synchronization of medical data

ECG (MedEx MCA-09012A) recordings were collected simultaneously with the BCG recordings. ECG (Mindary M7 Expert P4-2S and JJET LEAD_EPG) recordings were also acquired for the synchronization between the BCG recordings and the echocardiograms (Mindary M7 Expert P4-2S) or the intracardiac pressure curves (JJET LEAD_EPG). The BCG signal, echocardiography, and intracavitary pressure recording devices were synchronized with the ECG signal. This allowed for time alignment for the BCG signal, echocardiography, and intracavitary pressure measurements based on the ECG.

#### Verification of the correspondence between BCG and ECG recordings

The ECG and BCG recordings of 101 participants with normal cardiac function (Table 1) were synchronously acquired. A 15-s continuous and stable recording was taken for each subject, to compare the correspondence between the BCG and ECG recordings in participants in terms of sinus rhythm, APBs, or VPBs.

#### BCG-based cardiac cycle phase categorization

Six participants underwent intracardiac pressure monitoring by percutaneous femoral artery puncture with digital subtract angiography at the cardiac catheterization room of the Department of Cardiology, Zhongnan Hospital of Wuhan University. Two individuals had VPBs, two had supraventricular tachycardia, and two underwent coronary angiography. The tube (Name, Camdale pig5f.) was used to simultaneously measure the left ventricular pressure (LVP) and right ventricular pressure (RVP), respectively. The left ventricular intracavitary angiography tube was withdrawn to the aortic root, and the aortic pressure (AoP) curve was recorded. Simultaneously, the BCG recordings, ECG recordings, and echocardiograms were obtained. Then, the LVP and RVP values were time-aligned by the synchronous acquisition of ECG and BCG recordings.

### Validation of BCG-Based cardiac cycle phases

#### Hemodynamic parameter measurement by TDI

The TDI^25,29^ and BCG recordings of 51 participants (Supplementary Table S1) were obtained simultaneously. An 8-s continuous and stable recording was taken for each participant. The TDI data were measured by two-color Doppler flow imaging certified doctors and the average was considered as the outcome measure. The measured data included the interval from mitral valve closure to aortic valve opening (IVCT), the interval from aortic valve opening to aortic valve closing (LVET), and the interval from aortic valve closing to mitral valve opening (IVRT).

#### Data collection in resting state and after exercise

The BCG recordings, ECG recordings, and M-mode echocardiograms of 11 participants with normal cardiac function (Supplementary Table S2) were obtained simultaneously at a resting state and after 30 quick squats. The cardiac output (CO, L/min) was calculated by CO = *SV* ∙ *HR*, where *SV* (L/min) represents the stroke volume and *HR* (beats/min) represents the heart rate. *SV* and *HR* were obtained by taking the average value of three cardiac cycles from the M-mode echocardiograms, as calibrated by two doctors certified in color Doppler flow imaging, The IJ amplitude was calculated by taking the amplitude difference between the points I and J. These data were then used to analyze the relationship between the BCG waveform and the myocardium contractility.

### The usefulness of BCG-Based cardiac cycle phases

#### BCG recordings of patients with CHF

A total of 61 patients with CHF were included in this analysis, and all of them received standard medications including cardiotonic agents, diuretics, and arterial dilators. From the 61 participants, we collected the BCG signals during the initial phase of treatment. BCG recordings were continuously taken to observe the variation in the waveform. Simultaneously, Echocardiogram reports were collected. The variations in BCG waveforms and indicators of myocardial contractility were analyzed.

Classification of BCG-derived cardiac parameters for cardiac function evaluation.

#### Data collection and preprocessing

BCG data were collected from a total of 162 participants, including 101 participants with normal cardiac function and 61 participants with CHF. The BCG-based cardiac cycle phase categorization was utilized to extract time and amplitude cardiac parameters, especially the HI interval, IM interval, MN interval, HI amplitude, IJ amplitude, MN amplitude, HI slope, IJ slope, and MN slope (Table S3). To obtain these parameters, a dynamic time warping^30^ fusion analysis was performed on the cardiac cycle.This analysis yielded a total of 541 sets of samples, with each set comprising the aforementioned parameters, before training the dataset, we also conducted standardization processing on the dataset.

#### Training and testing the classification models

Six distinct classification models were employed in this study, namely SVM, KNN, DTC, LR, RF and XGBoost. These models were trained and evaluated using a dataset comprising nine parameters. The total sample size consisted of 541 groups. To enhance model performance, an extensive grid search was conducted on both the training and validation sets to identify the optimal hyperparameters. Detailed information on the hyperparameters for each model is provided in Supplementary Table S4. Subsequently, the models underwent rigorous evaluation using five-fold cross-validation, with key metrics such as accuracy, area under the curve (AUC), sensitivity, specificity, and F1 score used to assess their efficacy.

### Statistical analysis

Data analysis was conducted using MATLAB (R2020b) and a statistical package for the social sciences (SPSS) programming platform. Time intervals and amplitudes obtained by BCG or tissue Doppler echocardiographic techniques were expressed as mean ± standard deviation. To evaluate the accuracy of the measurements, the median value of the absolute errors, spearman's rank correlation coefficient r, and *R*^2^ were computed using the equations outlined in Reference ^31^. A Pearson correlation^32^ plot was generated to visualize the strength and direction of the correlation between the variables. To assess the clinical value of BCG-based cardiac cycle phase categorization, we analyzed the BCG parameters ranges of distribution ranges from participants with normal cardiac function and heart failure patients. We used the paired sample t-test to compare the indices, and a p-value less than 0.05 was considered indicative of significant differences across the measured parameters.

### Supplementary Information

Supplementary Information.

## Data Availability

All data needed to evaluate the conclusions in the study can be found in the paper and Extended data. The original data are available from the corresponding authors (Z.Li and Z.Lu) on reasonable request.
