# Supplementary material for: Non-contact assessment of cardiac physiology using FO-MVSS-based ballistocardiography: a promising approach for heart failure evaluation
Source: Sci Rep. 2024 Feb 8;14:3269. doi: 10.1038/s41598-024-53464-8 (PMC10853251; doi:10.1038/s41598-024-53464-8)
Supplement: Supplementary file 1 — Supplementary Information. [file 41598_2024_53464_MOESM1_ESM.pdf]

Non-Contact Assessment of Cardiac Physiology Using FO-MVSS-Based Ballistocardiography: A Promising Approach for Heart Failure Evaluation

Jing Zhan[1,2+], Xiaoyan Wu[4,5+], Xuelei Fu[1], Chenze Li[4,5], Ke-Qiong Deng[4,5], Qin Wei[1], Chao Zhang[4,5], Tao Zhao[1,2], Congcong Li[1,2], Longting Huang[1], Kewei Chen[1,2], Qiongxin Wang[4,5], Zhengying Li[1,2,3,6*], Zhibing Lu[4,5*]

[1] *Hubei Key Laboratory of Broadband Wireless Communication and Sensor Networks, School of Information Engineering, Wuhan University of Technology, Wuhan 430070, Hubei, China*
[2] *National Engineering Research Center of Optical Fiber Sensing Technology and Networks, Wuhan University of Technology, Wuhan 430070, Hubei, China*
[3] *State Key Laboratory of Silicate Materials for Architectures, Wuhan University of Technology, Wuhan 430070, Hubei, China*
[4] *Department of Cardiology, Zhongnan Hospital of Wuhan University, Wuhan, 430071, Hubei, China*
[5] *Institute of Myocardial Injury and Repair, Wuhan University, Wuhan, 430071, Hubei, China*
[6] *State Key Laboratory of Advanced Technology for Materials Synthesis and Processing, Wuhan University of Technology, Wuhan, 430070, Hubei, China*

[*]Corresponding authors: zhyli@whut.edu.cn, luzhibing222@163.com
Contributing authors: jingzhan@whut.edu.cn, wuxiaoyan299@aliyun.com, xlfu@whut.edu.cn, lichenze@whu.edu.cn, dengkeqiong@whu.edu.cn, qinwei@whut.edu.cn, zhangchao@znhospital.cn, taozhao@whut.edu.cn, congcongli@whut.edu.cn, huanglt08@whut.edu.cn, ckw@whut.edu.cn, qiongxin-wang@whu.edu.cn,
[+]These authors contributed equally to this work

Funding: This work was supported by the National Natural Science Foundation of China (61735013 to Z.Li and 82070425 to Z.Lu).

Author Disclosures: All authors have reported that they have no relationships relevant to the contents of this paper to disclose.

Supplementary Method

Study population and protocol

The study population was represented by participants that were recruited across the four distinct stages (Supplementary Fig. S7).

In the first stage, 10 participants with normal cardiac function (age range: 20-25 years, 7 males, 3 females) were selected from 111 participants recruited with normal cardiac function to assess the stability and consistency of the BCG signals obtained using the FO-MVSS. Eligible participants were required to have no history of cardiovascular or respiratory diseases and demonstrate the ability to adhere to the experimental instructions. Participants who did not meet these criteria were excluded from the study. The specific exclusion criteria were as follows: (1) Participants with tremors, such as Parkinson's disease. (2) Participants are unwilling and unable to adhere to the following instructions and cooperate during the experiment.

In the second stage, the study involved participants with normal cardiac function to analyze the correlation between BCG and cardiac motion. In the experiment, the inclusion criteria were as follows: (1) participants without any significant cardiac, respiratory, or physical conditions or discomfort were selected. (2) Participants were required to remain calm during the recordings and comply with the experimental instructions. There were 101 participants (age range: 20-78 years, 55 males, 46 females) who were selected from the 111 participants recruited with normal cardiac function in this stage. Simultaneous BCG and electrocardiogram (ECG) recordings were obtained to investigate the correlation between the BCG waveforms and cardiac motion.

In the third stage, the study aimed to analyze the relationship between BCG waveforms and the phases of the cardiac cycle and validated the effectiveness of BCG-based cardiac cycle phases categorization. The first experiment in this third stage involved 8 patients selected from the 111 participants recruited with normal cardiac function. These patients were required to undergo cardiac catheterization and accept simultaneous collection of BCG signals. It was essential for these patients to fully cooperate with experimental instructions. Participants with incomplete experimental records were

excluded. Finally, a total of 6 patients (age range: 33-55 years, 4 males, 2 females) were included in this analysis. We collected the BCG, ECG, and intracavitary pressure signals to analyze their relationship with the phases of the cardiac cycle. The comparison of the characteristics from the BCG waveforms with the different phases of the cardiac cycle was achieved, to hypothesize a BCG-based cardiac cycle staging method.

In the second and third parts of this stage, our study involved medical validation and incorporated two sub-experiments to assess the accuracy of the BCG-based cardiac cycle phase categorization.

(a) TDI validation. There were 51 participants who were randomly selected from the 111 participants recruited (age range: 25-45 years, 41 males, 10 females) and underwent TDI classification[21] to extract cardiac time parameters corresponding to different phases of the cardiac cycle. The extracted cardiac time parameters were then compared with the corresponding cardiac time intervals[21] obtained from the BCG signals to validate the accuracy of the BCG-based cardiac phase categorization.

(b) Waveform amplitude and CO association validation. For this analysis, 11 young participants (age range: 20-30 years, 11 males) were selected from the 111 recruited participants with normal cardiac function. Participants were required to provide rest and exercise BCG and M-mode Doppler ultrasound data. The analysis focused on investigating the association between the changes in waveform amplitudes within the ejection phase of the BCG signal and the cardiac output. The aim was to further validate the accuracy of the BCG-based cardiac cycle phase categorization.

In the fourth stage, 61 patients with CHF (age range: 48-76 years, 27 males, 34 females) were recruited for evaluating the potential of the BCG-based cardiac cycle phases. BCG recordings were collected, and the association between the time parameters and amplitude parameters involved in the BCG-based cardiac cycle phase categorization and the left ventricular ejection fraction was evaluated. The aim was to provide a pathological perspective and a preliminary analysis of the clinical application of the BCG-based cardiac cycle phase categorization.

FO-MVSS circuit. The microcontroller (STM32H743) of the FO-MVSS (Fig. 1) was set at a sampling

frequency of 1024 Hz. Firstly, the optical signal modulated by the vibration was converted into an electrical signal using a photodetector (PD). Secondly, the electrical signal was sent to pre-amplifier 1, which performed high-pass filtering (second-order active high-pass filter, cut-off frequency: 0.1 Hz) with 100 times amplification. The output contained both the respiratory and heartbeat signal components. Thirdly, the signal was subjected to further high-pass filtering (second-order active high-pass filter, cut-off frequency: 0.3 Hz) with 20 times amplification in pre-amplifier 2. Then, the output was converted into a digital signal by an A/D convertor. Finally, the processed digital signal was transmitted to the computer.

4

Supplementary Tables

Table S1. Population characteristics (*n*=51) in verification of correspondence between BCG recordings and TDI

Characteristics	Mean Value	SD	Maximum	Minimum
**Age (years)**	35.1	10.2	45.0	25.0
**Male (N, percentage)**	41, 80%	—	—	—
**Height (cm)**	171.2	8.1	189.0	153.0
**Body weight (kg)**	65.7	11.3	77.0	54.0
**BMI (kg/m^2)**	22.3	3.1	25.0	19.0
**HR (beats/min)**	73.0	12.2	85.1	60.9
**EF (%)**	66.3	7.2	80.0	53.1
**SV (ml)**	71.6	16.5	106.8	43.1
**CO (L/min)**	5.2	1.6	9.4	3.0

BMI, body mass index; HR, heart rate; EF, ejection fraction; SV, stroke volume; CO, cardiac output.

5

Table S2. Population characteristics (*n*=11) in data collection at the resting rate and after exercise

Characteristics	Mean Value	SD	Maximum	Minimum
**Age (years)**	24.6	5.6	30.0	20.0
**Male (N, percentage)**	11,100%	—	—	—
**Height (cm)**	171.4	2.0	175.0	169.0
**Body weight (kg)**	61.5	7.7	76.0	48.0
**BMI (kg/m^2)**	20.9	2.5	24.8	16.4
**HR (beats/min)**	72.2	13.0	105.0	55.0
**EF (%)**	63.4	4.9	73.7	56.5
**SV (ml)**	69.8	12.0	90.1	43.1
**CO (L/min)**	5.0	0.8	5.9	3.1

BMI, body mass index; HR, heart rate; LVEDV, left ventricular end-diastolic volume; LVESV, left ventricular end-systolic volume; EF, ejection fraction; SV, stroke volume; CO, cardiac output.

6

Table S3. The distribution of cardiac time intervals and amplitudes parameters measured by BCG in the control group and CHF patients.

Characteristics	BCG waveform	Physiological significance
**HI interval (ms)**	H to I point interval	IVCT
**IM interval (ms)**	I to M point interval	LVET
**MN interval (ms)**	M to N point interval	IVRT
**HI amplitude (mV)**	the amplitude of H to I point	-
**IJ amplitude (mV)**	the amplitude of I to J point	the variation of it correlates linearly with CO variation
**MN amplitude (mV)**	the amplitude of M to N point	-
**HI slope**	the slope of H to I point	-
**IJ slope**	the slope of I to J slope	-
**MN slope**	the slope of M to N slope	-

IVCT, isovolumetric contraction time; LVET, left ventricular ejection time; IVRT, isovolumetric relaxation time.

7

Table S4. Overview of Hyperparameters for Classification Models in the Study

Model	Hyperparameters	Value
**SVM**	Cost	1
	Gamma	scale
	Kernel	linear
**KNN**	N_neighbors	2
	P	1
	Weights	distance
**DTC**	criterion	gini
	Max_depth	3
	Min_samples_leaf	4
	Min_samples_split	2
**Logistic regression**	C	100
	Penalty	L2
**Radom Forest**	Max_depth	4
	Min_samples_leaf	3
	Min_samples_split	3
	N_estimators	50
**XGboost**	Learning_rate	0.1
	Max_depth	3
	N_estimators	100

LR, Logistic regression. SVM, support vector machines; KNN, K-nearest neighbor;DTC, Decision tree. RF, Radom forest. XGBoost, Extrme gradient boosting.P, the power parameter for the minkowski distance. C, the inverse of regularization strength, and higher values indicate weaker regularization.

8

Supplementary Figures

Figure S1

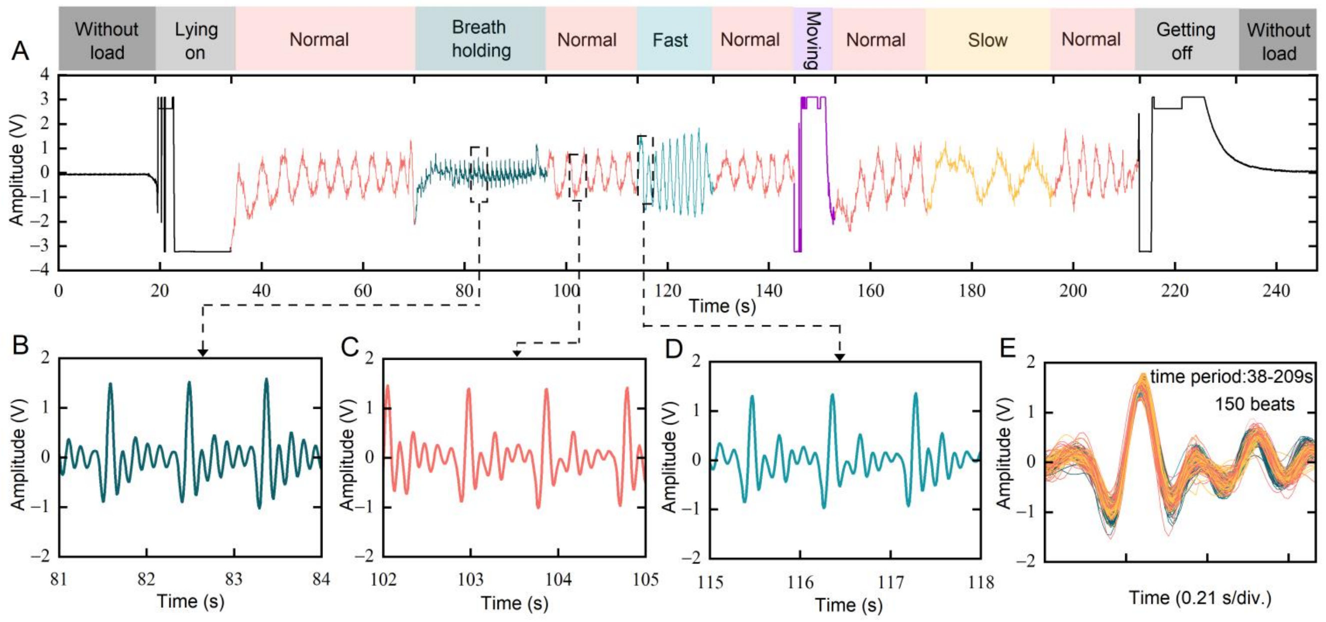

Fig. S1. Influence of respiratory rate on the waveform morphology of the extracted heartbeat vibration signal.

A, Output signal of pre-amplifier 1, which contains both the heartbeat and respiratory components. 0–18 seconds: unattended; 18–35 seconds: subject lying down on the FO-MVS; 35–68, 94–113, 129–145, 153–172, and 196–209 seconds: subject breathing normally (respiratory rate: 12–24 breaths/min); 70–93 seconds: subject holding their breath; 114–128 seconds: subject breathing rapidly (respiratory rate: >24 breaths/min); 172–196 seconds: subject breathing slowly (respiratory rate: <12 breaths/min); 143–155 seconds: subject moving; 209–240 seconds: subject getting off of the FO-MVS.

B–D, Heartbeat-induced vibration signals extracted during breath hold, slow breathing, and rapid breathing. **E,** Repeatability verification of the waveform morphology by superimposing 150 waveforms extracted from the signal in **A** (38–209 seconds).

Figure S2

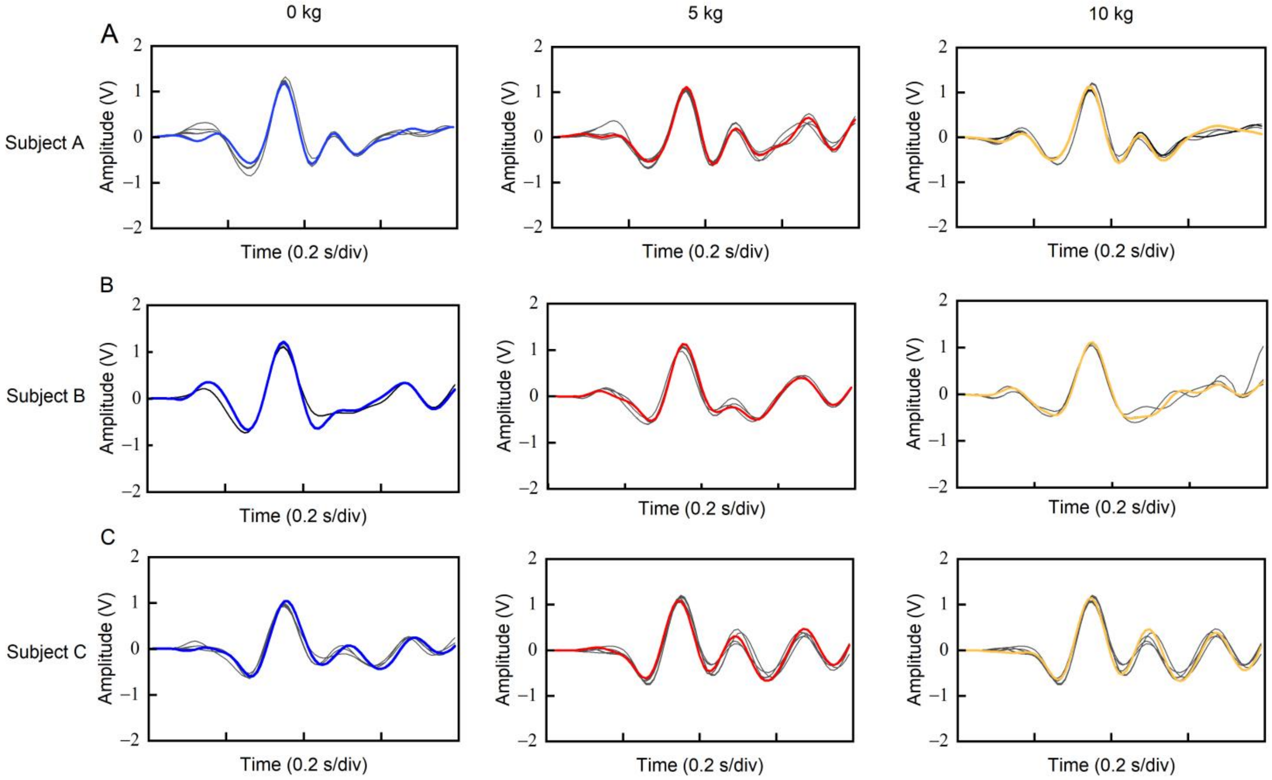

Fig. S2. Influence of load bearing on the amplitude of the waveform.

A-C, Ballistocardiography (BCG) waveforms of Subjects A to C bearing loads of different weights.

Figure S3

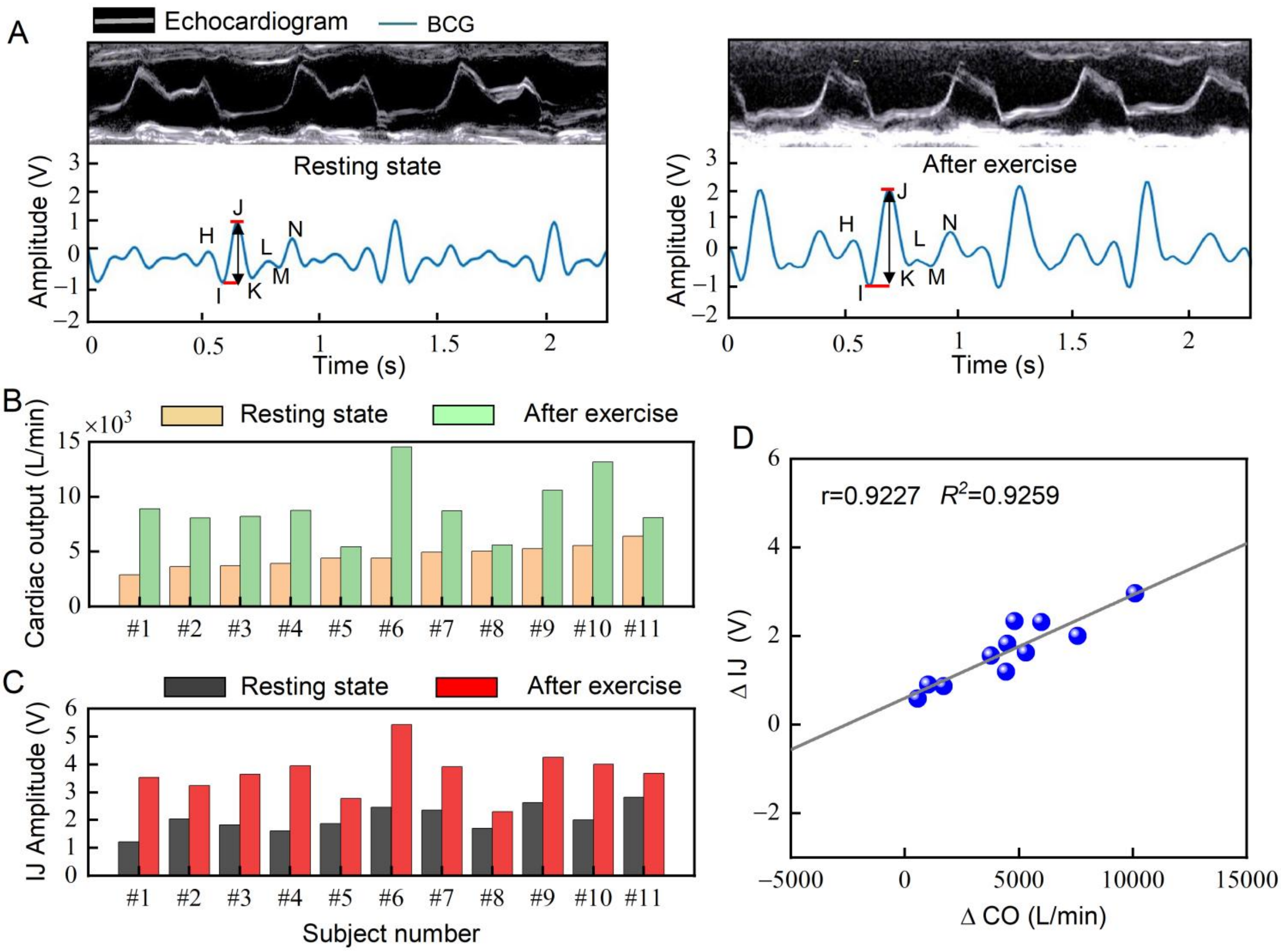

Fig. S3. Changes in the IJ amplitude and the cardiac output (CO) in resting state and after exercise.

A, Synchronously obtained BCG waveforms and M-mode echocardiograms of the mitral valve in one subject in the two different states.

B, The CO in the two different states.

C, The IJ amplitude in the two different states.

D, The linear relationship between the variation in the CO and the variation in the IJ amplitude.

Figure S4

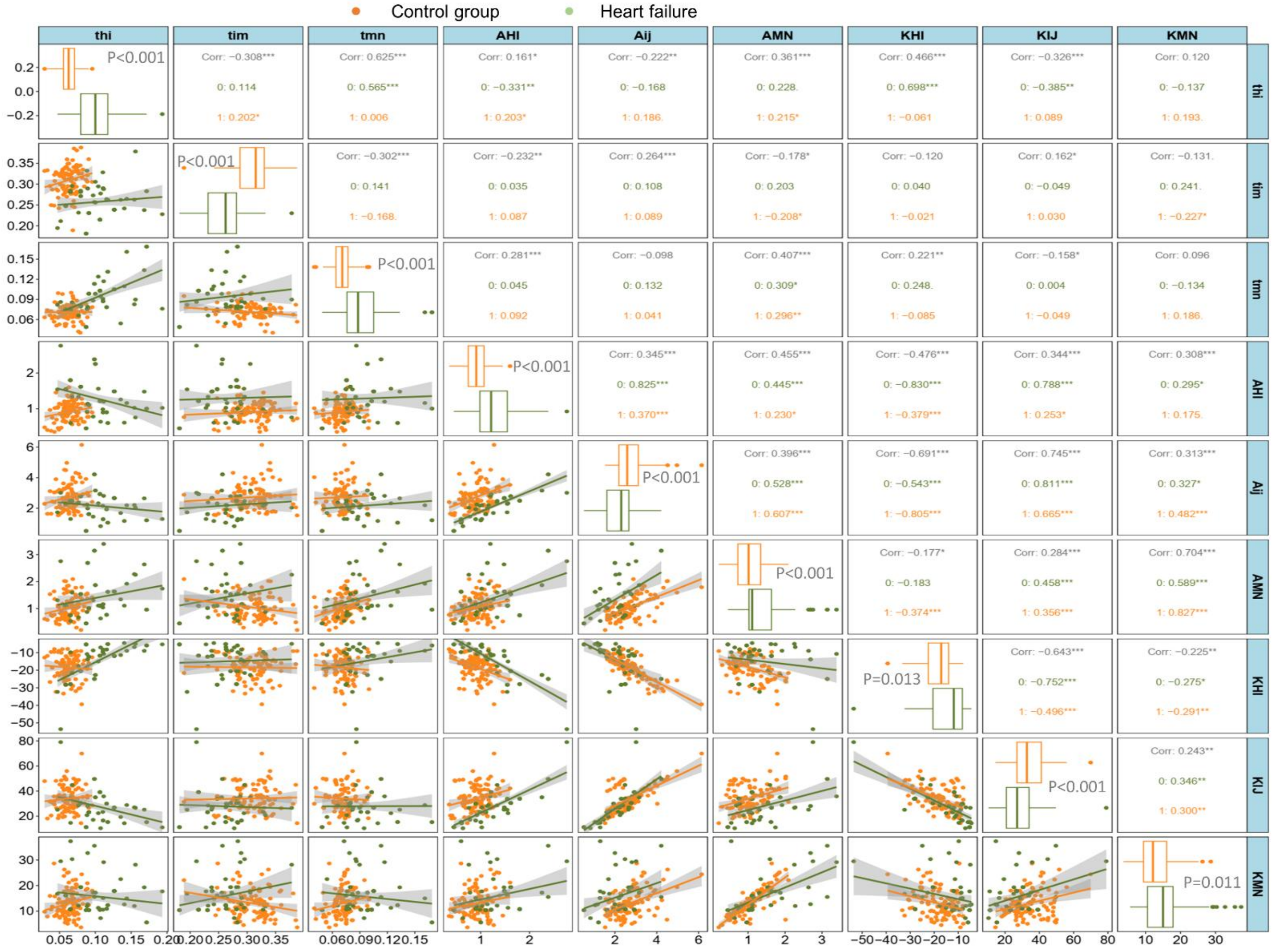

Fig. S4. Correlation Matrix and Feature Distributions. This figure illustrates the correlation matrix of the nine features in our dataset. The diagonal presents boxplots representing the distribution of each feature across different groups. In the lower-left section, scatter plots depict the pairwise correlation between features, providing insights into their associations. The upper-right section displays the corresponding correlation coefficients for each feature pair. This comprehensive visualization captures both the individual feature distributions and their interrelationships, offering a holistic view of the dataset's characteristics.

Figure S5

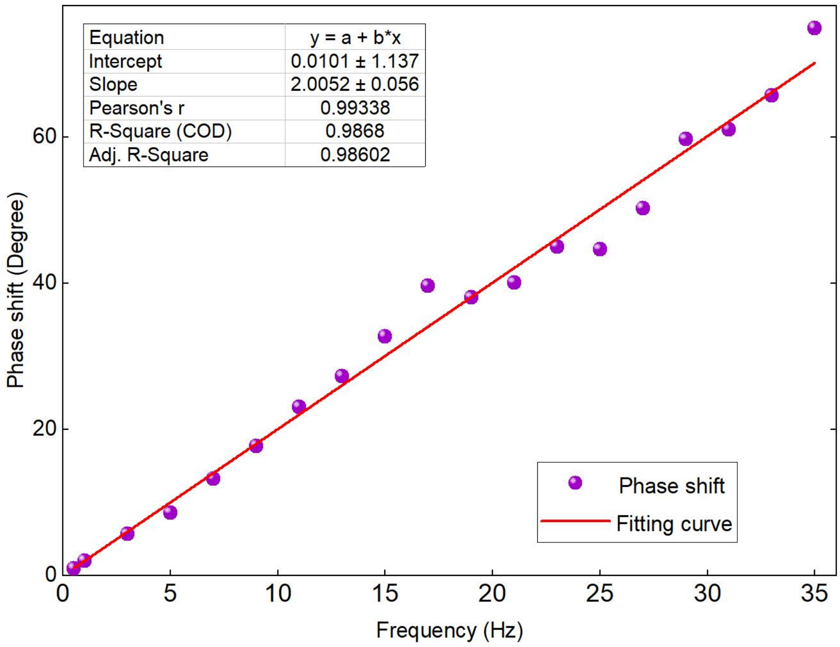

Fig. S5. Phase frequency response chart. As shown in the picture, the phase variation of the FO-MVSS's response shows an approximate linearity at different frequencies (0.5 – 35 Hz). This indicates that the group delay of the sensor is approximately constant. This result ensures that the signal from the sensor will not experience distortion within this frequency range.

Figure S6

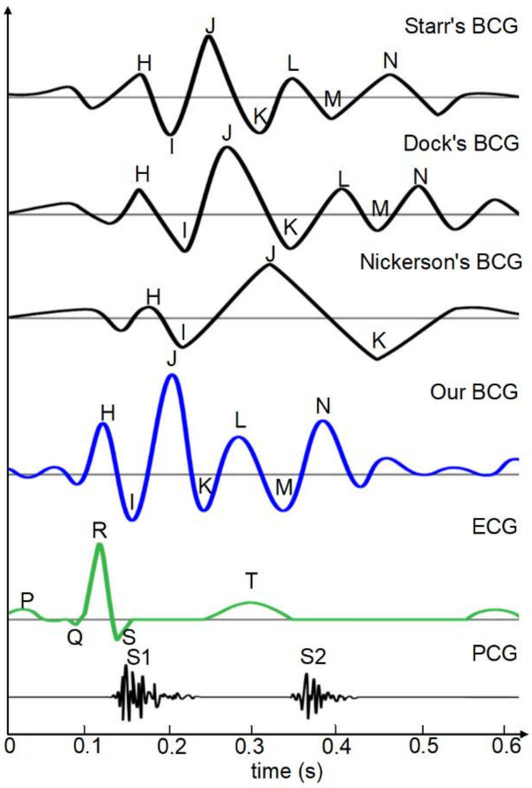

Fig. S6. Comparison chart of BCG and medical data at different periods[6]. The figure displays signals from a normal participant with a heart rate of 75 (beats/mins). From top to bottom, they are Starr's BCG[7], Dock's BCG[8], Nickerson's BCG[9], BCG obtained with FO-MVSS as described in this paper, as well as ECG and PCG signals. It can be observed from the figure that due to the high sensitivity and relatively flat amplitude-frequency characteristics of FO-MVSS in the BCG signal frequency range, the coupling between the BCG waveform studied in this paper and the ECG is closer, and the restored waveform is more detailed and rich.

Figure S7

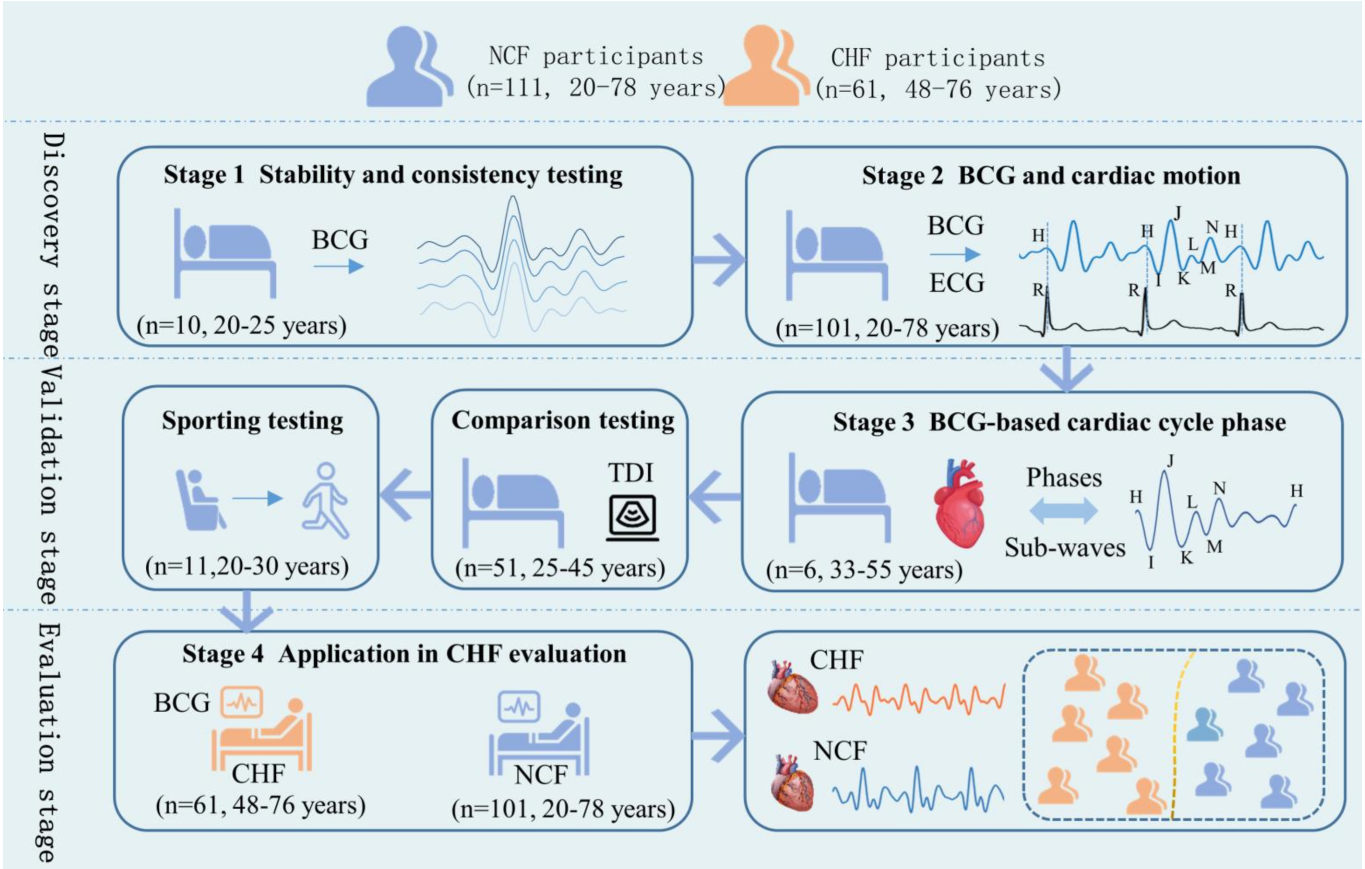

Fig. S7 Flow diagram of the study population and protocol. The flowchart illustrates the four stages of this study protocol. Collectively propose and validate the accuracy and potential clinical value of a BCG-based cardiac cycle phase categorization. NCF, normal cardiac function; CHF, congestive heart failure; Pre, pre-treatment; Post, post-treatment; BCG, ballistocardiography.
